# Management of Advanced Cutaneous Squamous Cell Carcinoma over the Last Decade: A Single-Centre Retrospective Study

**DOI:** 10.3390/curroncol33080449

**Published:** 2026-07-27

**Authors:** Ramon Staeger, Leandra Gioia Ehrat, Nicole Kamber, Reinhard Dummer, Mirjam C. Nägeli, Egle Ramelyte

**Affiliations:** 1 Department of Dermatology, University Hospital Zurich, 8091 Zurich, Switzerland; 2 Medical Faculty, University of Zurich, 8032 Zurich, Switzerland; 3 Department of Dermatologie, Kantonsspital Aarau, 5000 Aarau, Switzerland

**Keywords:** cutaneous squamous cell carcinoma, anti-PD1, immunotherapy, real-world evidence, chronic lymphocytic leukemia

## Abstract

**Introduction:** Cutaneous squamous cell carcinoma (cSCC) is one of the most common skin cancers, with a subset progressing to locally advanced (laSCC) or metastatic (mSCC) stages. The introduction of anti-PD1 immunotherapy has transformed treatment, but real-world data remain limited, particularly in immunosuppressed patients. **Methods:** This single-centre, retrospective study included 189 patients with advanced cSCC treated between 2012 and 2022. Demographic, clinical, and treatment data were analyzed to assess clinical management and outcomes before and after the introduction of anti-PD1. **Results:** Among the 189 patients, 72.5% were male, with a median age of 79 years. Overall, 86 patients presented with laSCC and 103 with mSCC. In 100 patients, a preceding primary cSCC was documented, and its complete resection (R0) was associated with significantly better overall survival (OS) after diagnosis of advanced disease (*p* < 0.001). Immunosuppressed patients, including organ transplant recipients and those with chronic lymphocytic leukemia (CLL), had significantly reduced OS (*p* = 0.017 and *p* = 0.0059, respectively). First-line treatment prior to 2018 predominantly involved surgery and radiotherapy. Following the introduction of anti-PD1 therapy, its use increased rapidly in both first- and second-line settings. From 2018 onward, the number of advanced cSCC cases discussed at the multidisciplinary tumorboard increased approximately threefold. Median OS was significantly longer for mSCC patients treated in the post-2018 era (*p* = 0.025), while the survival disadvantage of CLL patients compared to non-CLL patients widened, suggesting limited benefit from advances in systemic therapy in this subgroup. Best overall response to first-line anti-PD1 correlated significantly with OS, with complete responders achieving a 1-year progression-free survival of 83.3%. **Conclusions:** The introduction of anti-PD1 has demonstrated improved survival outcomes in advanced cSCC, though significant challenges remain for immunosuppressed patients, particularly those with CLL and solid organ transplant recipients. Future research should focus on optimizing treatment for these high-risk groups, therapeutic sequencing, and the role of perioperative (neoadjuvant and adjuvant) immunotherapy strategies.

## Simple Summary

Cutaneous squamous cell carcinoma is among the most common skin cancers and is usually cured by surgical excision. A subset of tumors, however, becomes locally advanced or metastatic and requires multidisciplinary management. The introduction of immune checkpoint inhibitors targeting PD-1 has substantially changed the treatment of these advanced cases, but real-world data remain limited, particularly for patients excluded from registration trials. We analyzed 189 patients with advanced cutaneous squamous cell carcinoma treated at a Swiss tertiary referral centre between 2012 and 2022. From 2018 onwards, immunotherapy was rapidly adopted in both first- and second-line treatment, and survival improved in patients with metastatic disease. In contrast, organ transplant recipients and patients with chronic lymphocytic leukemia continued to show poor outcomes. As immunotherapy now moves into the neoadjuvant and adjuvant setting, these immunosuppressed populations remain underrepresented in trials and require dedicated prospective studies to guide their treatment.

## 1. Introduction

Cutaneous squamous cell carcinoma (cSCC) is the second most common skin cancer, with a rising incidence, particularly in the aging population [1]. While most cSCCs are treated effectively with surgical excision [2], a subset of cases progresses to advanced tumors, encompassing locally advanced (laSCC) and metastatic (mSCC) cases [3,4]. MSCC includes loco-regional (with in-transit or lymphonodal metastasis) and distant metastatic cSCC [3]. LaSCC are of large size, may infiltrate beyond the subcutaneous tissue into nerves, muscles or bone, and may have a history of multiple local recurrences, so that treatment with surgery or radiotherapy shows lower chances of cure and may result in unacceptable complications, morbidity or deformity [3].

Advanced cSCC is associated with significant morbidity and mortality, especially in patients with immunosuppressive conditions, such as solid organ transplant recipients (SOTR) [5] and those with hematological malignancies like chronic lymphocytic leukemia (CLL) [6,7]. In SOTR, this risk is driven by long-term iatrogenic immunosuppression, in particular azathioprine, which sensitizes DNA to UVA and produces a characteristic mutational signature [8], and calcineurin inhibitors, which impair immune surveillance and DNA repair [9]. In CLL, the increased risk relates to disease-associated immune dysfunction rather than to drug treatment.

The management of advanced cSCC has long relied on surgery and radiotherapy as major cornerstones [4,10]. However, therapeutic options have expanded over the last decade, especially with the introduction of immune checkpoint inhibitors (ICIs), such as anti-PD1 antibodies [11]. PD1-blockade has shown great efficacy in cSCC, even in an elderly population [12,13], likely due to the heavy mutational burden originating from UV damage in the malignant keratinocytes [8,14]. In patients with advanced and metastatic cSCC, anti-PD1 showed high response rates (between 44 and 46.1%), with 13–16.1% of patients achieving a complete response [12,13]. Median duration of response was not reached, with an estimated proportion of 87.8% (95% CI between 66.7 and 95.9%) of responses exceeding 12 months [12,13].

The European Medicines Agency (EMA) approved cemiplimab, the first anti-PD1 agent for advanced cSCC, mid-2019, marking a significant milestone in cSCC treatment [15]. Real-world evidence on the impact of these newer therapies remains limited, particularly in special populations, such as the immunosuppressed [16,17,18].

The aim of this single-centre, retrospective study was to assess the real-world management of advanced cSCC over the past decade, with a focus on evolving treatment strategies, particularly the adoption of ICIs. The study seeks to describe the demographic and clinical characteristics of the patient population and compare treatment outcomes before and after the introduction of ICIs in a tertiary skin cancer centre in Switzerland.

## 2. Materials and Methods

In this single-centre retrospective cohort study, patients with cSCC discussed at the multidisciplinary skin cancer tumorboard at the University Hospital Zurich, Switzerland, between April 2012 and October 2022, were included. The University Hospital Zurich is a tertiary referral centre, and its skin cancer tumorboard is the primary forum for the discussion of advanced cSCC, with the ENT department as a permanent member. Patients were identified through the electronic health record system by a digital search of the complete skin cancer tumorboard list, which records the diagnosis for each presented case, across the entire study period. Demographic, clinical, and pathological data was extracted from patient records.

Statistical analyses were performed by using R (version 4.4.1). Categorical variables were summarized as frequencies, and Fisher’s exact test was used to assess differences. Continuous variables were summarized as median and range or mean and standard deviation (SD) and analyzed by Wilcoxon rank test. Progression-free survival (PFS) and overall survival (OS) were calculated from initiation of treatment until progression or death from any cause, respectively. For patients with no events at time of data collection, the date of last contact was used for censoring. Time-to-event analyses were visualized by Kaplan–Meier (KM) curves. Censored observations are indicated by vertical tick marks in the KM curves. Survival differences were tested with the log-rank test. A *p*-value of <0.05 was considered as statistically significant.

## 3. Results

### 3.1. Patient Demographics

A total of 189 patients with advanced cSCC were included, of which 72.5% were male and 27.5% female (Table 1). At diagnosis of advanced cSCC, the median age was 79 years (range 37–99). Overall, 86 (45.5%) presented with laSCC and 103 (54.5%) with mSCC. In 100 patients, the advanced cSCC arose from a previously diagnosed primary non-advanced cSCC, while the remaining patients presented with advanced disease at first diagnosis.

**Figure 1:**
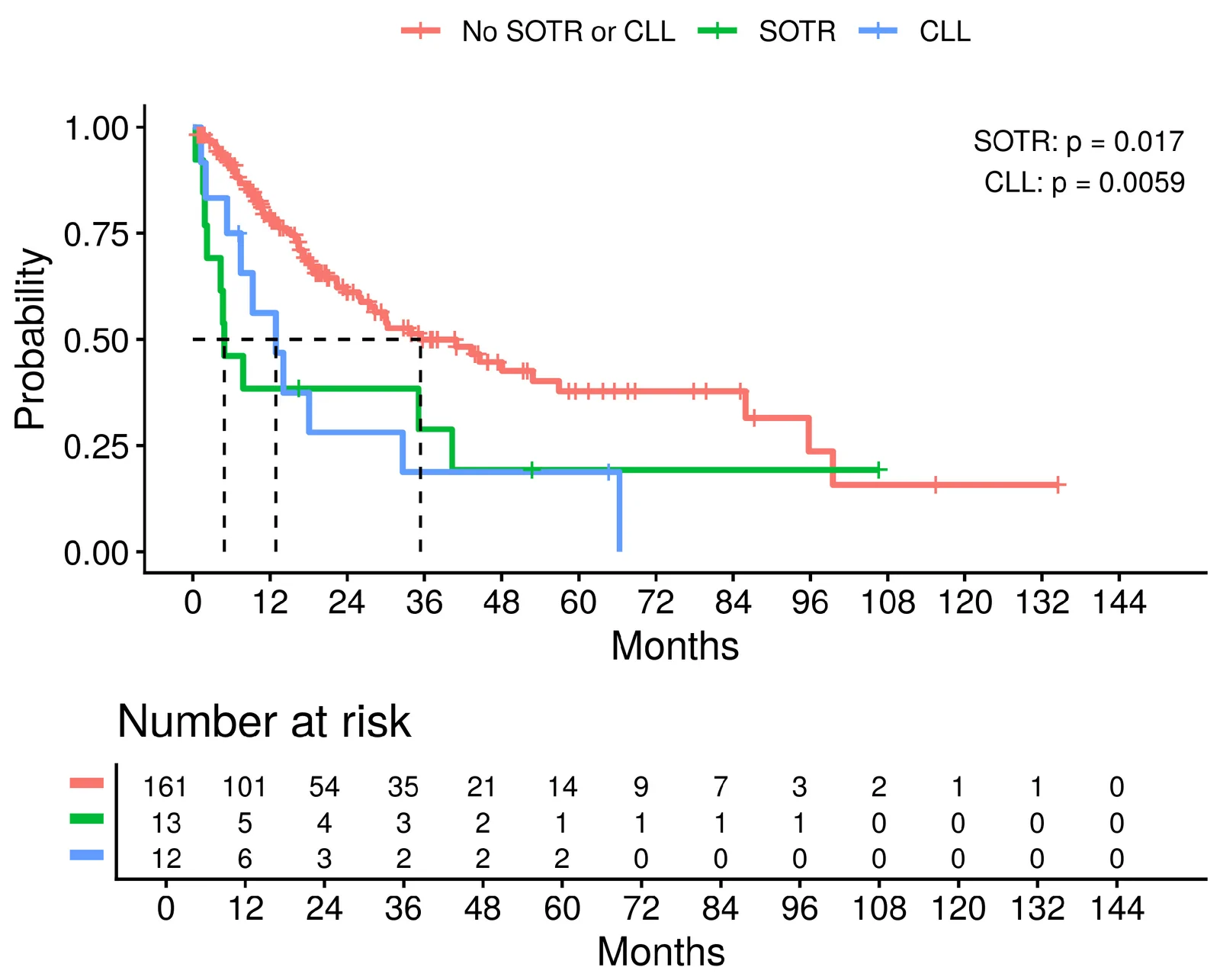
Overall survival (OS) by immunosuppression status in patients with advanced cSCC (*p*-values from pairwise log-rank tests versus patients with neither condition). SOTR, solid organ transplant recipients; CLL, chronic lymphocytic leukemia.

The majority of patients had a history of significant skin cancerogenesis: At least five actinic keratoses (AK) were documented in 86 patients (45.5%), 109 (57.7%) reported at least one previous cSCC, and 89 (47.1%) at least one previous basal cell carcinoma (BCC). A subgroup of patients were immunosuppressed: 13 patients (6.9%) were solid organ transplant recipients (SOTR), of which five were kidney, four heart and four lung transplantations. Furthermore, relevant pre-existing comorbidities were found in 37 patients (19.6%), including 12 patients (6.3%) with CLL, 13 patients (6.9%) with Non-Hodgkin Lymphoma (NHL), and eight patients (4.2%) with other hematological malignancies (incl. CML, myelodysplastic and myeloproliferative syndrome). An additional three patients had a chronic viral infection (HIV or HCV), and one patient had Xeroderma pigmentosum. Patients with an advanced cSCC who were SOTR or were suffering from CLL had a significantly reduced overall survival (OS) (*p* = 0.017 and 0.0059, respectively) (Figure 1). The median OS for SOTR patients was 4.9 months (95% CI: 2.17-NR) after diagnosis of advanced cSCC, and 12.9 months (95% CI: 7.43-NR) for patients with CLL. Among the 12 CLL patients (nine mSCC, three laSCC), first-line treatment was surgery alone in five, surgery combined with radiotherapy in four, anti-PD1 in two, and radiotherapy in one patient. Anti-PD1 was administered in four patients across all lines, with one complete and one partial response; the two non-responders progressed within 2.7 and 3.2 months. Among the 13 SOTR (five kidney, four heart, four lung), first-line treatment was surgery alone in eight, surgery combined with radiotherapy in three, and radiotherapy in two patients. Anti-PD1 was administered in only two SOTR (one kidney and one lung recipient, both in third-line), with this treatment being frequently withheld because of the risk of transplant rejection.

### 3.2. Primary Tumors Giving Rise to Advanced cSCC

**Figure 2:**
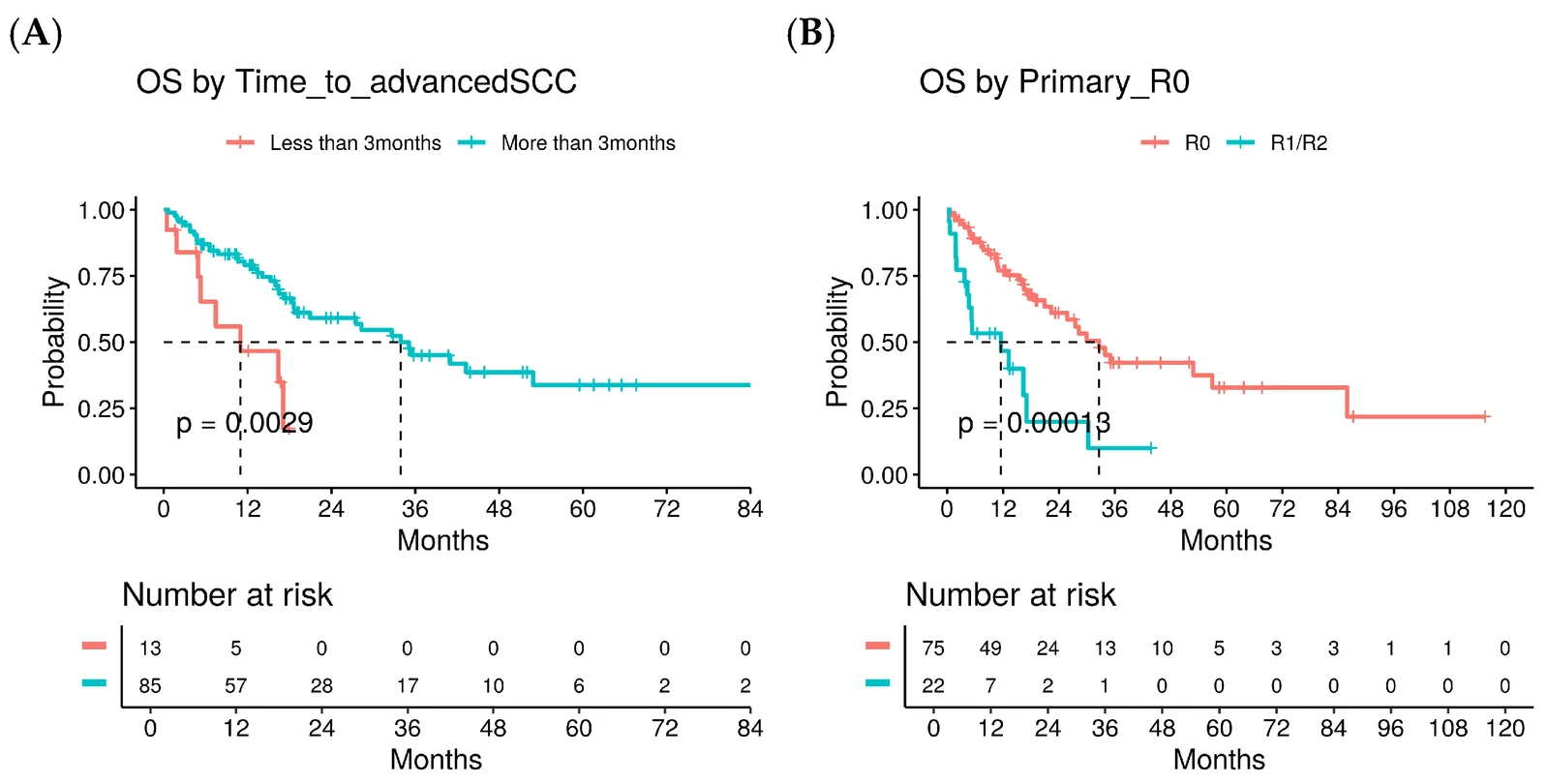
(**A**) Overall survival (OS) for patients who progressed from primary to advanced cSCC in less than 3 months. (**B**) OS after diagnosis of advanced cSCC by resection status of the primary tumor. R0, complete resection with histologically tumor-free margins; R1, microscopic residual tumor at the resection margin; R2, macroscopic residual tumor.

Of the 189 patients with advanced cSCC, 100 were previously diagnosed with a primary non-advanced cSCC (Table 2). At diagnosis of the primary cSCC, the median age was 77 years (range 43–94). Primary tumors were most commonly located in the face (*n* = 63, 63.0%) and scalp (*n* = 18, 18.0%). A total of 35 tumors (35%) were localized in a high-risk anatomical area (incl. periocular, lip, cheek, temple, ear). The vertical tumor thickness was available in 55% of cases, at a median of 4 mm (mean 5.43 mm). In nine cases (9%), perineural invasion (PNI) was diagnosed by histology. Most primary tumors were treated by surgical excision (85%), of which 76.5% achieved complete resection with histologically tumor-free margins (R0) on the first attempt. The median time from diagnosis of primary to development of the advanced cSCC was 12.2 months (range 0.8–132). The time to advanced cSCC correlated with outcome: Patients with progression within 3 months of primary diagnosis showed a significantly worse OS (*p* = 0.0029) (Figure 2A).

Patients whose primary cSCC was surgically removed with histological confirmation of clear margins (R0 resection) had significantly longer OS following diagnosis of advanced stage cSCC (*p* < 0.001) (Figure 2B).

### 3.3. Locally Advanced cSCC

Of the 189 patients with advanced cSCC, 86 (45.5%) presented with a locally advanced tumor (Table 3). The median horizontal diameter was 36 mm (*n* = 40), and the median vertical thickness was 12.5 mm (*n* = 38), where available.

First-line treatment was in most cases surgery alone (*n* = 42, 48.8%) or combined with radiotherapy (*n* = 19, 22.1%), followed anti-PD1 (*n* = 14, 16.3%) and primary radiotherapy (*n* = 9, 10.5%). In cases approached by surgical treatment (*n* = 61), 39 (63.9%) achieved R0; however, 13 (21.3%) and four (6.6%) had a microscopic (R1) or macroscopic (R2) residual tumor, respectively. The median OS was 44.5 months (95% CI: 30.1-not reached).

### 3.4. Metastatic cSCC

Of the 189 patients with advanced cSCC, 103 (54.5%) presented with metastatic disease (Table 4). In the majority of cases (92.2%), the metastatic spread was limited to regional locations (i.e., in-transit and/or lymph node metastases). Distant metastases at diagnosis were reported in 7.8% of cases.

First-line treatment was most commonly combined surgery and radiotherapy (*n* = 45, 43.7%), followed by surgery alone (*n* = 27, 26.2%), anti-PD1 (*n* = 19, 18.4%) and primary radiotherapy (*n* = 7, 6.8%) (Table 4). Median OS was 23.7 months (95% CI: 18.0–56.9). Second-line treatment was required in 44 patients (42.7%), most commonly anti-PD1 therapy (*n* = 16, 36.4%).

### 3.5. Clinical Management of cSCC

**Figure 3:**
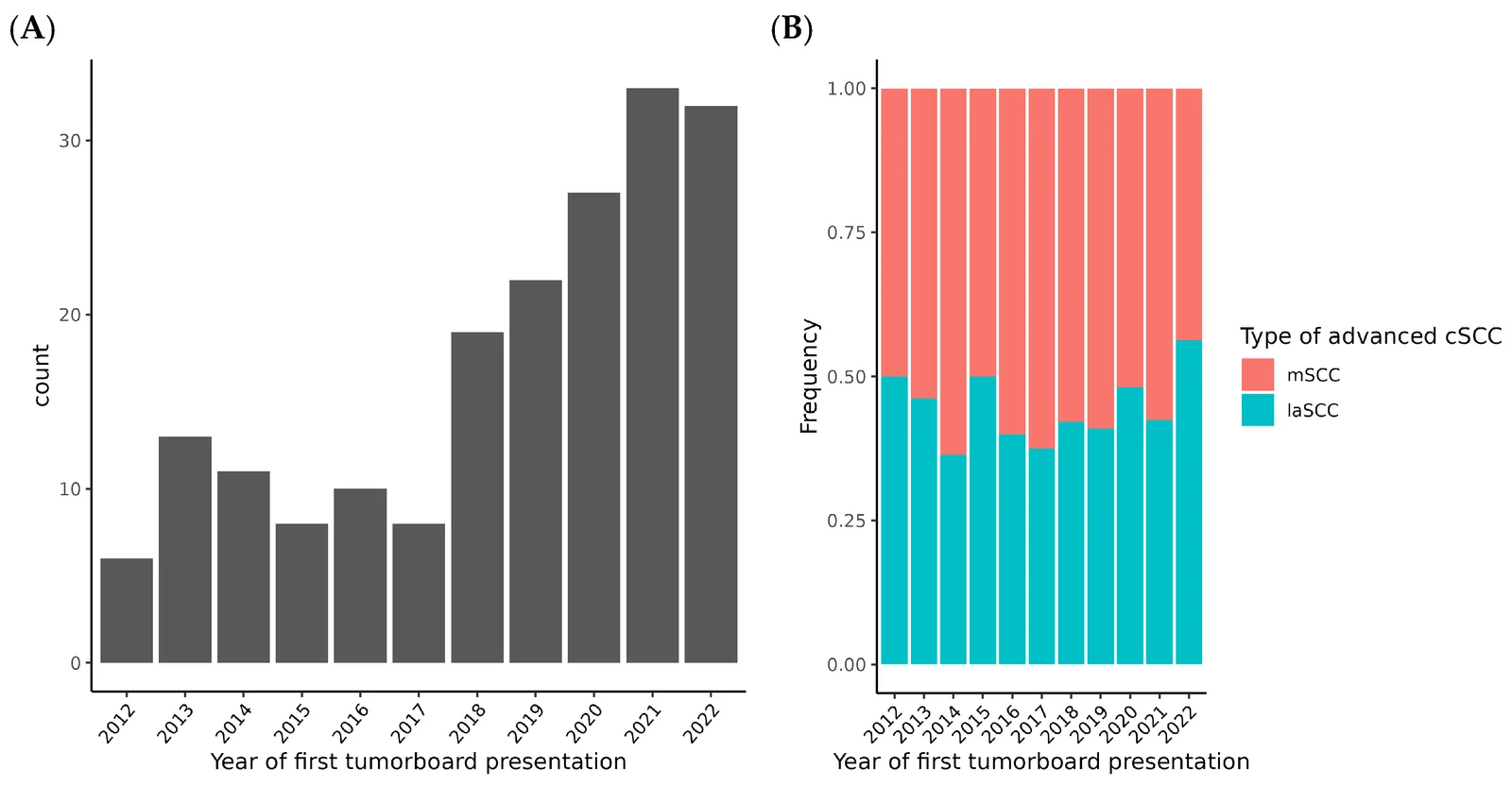
(**A**) Number of cases with advanced cSCC presented at the tumorboard by year. (**B**) Proportion of metastatic (mSCC) and locally advanced cSCC (laSCC) by year.

In the observed decade, the number of patients with cSCC discussed at the multidisciplinary tumorboard grew over fivefold, from six cases in 2012 to 33 cases in 2021 (Figure 3A). While numbers remained relatively stable from 2012 to 2017, there was a significant increase starting from 2018 (mean 9.3 cases/year versus 26.6 cases/year, *p* < 0.0001). Baseline characteristics did not differ significantly between the two eras (Supplementary Table S1). The distribution of laSCC and mSCC was similar throughout the years with roughly half of the cases being laSCC and mSCC, respectively (Figure 3B), indicating a stable case composition over the observation period.

**Figure 4:**
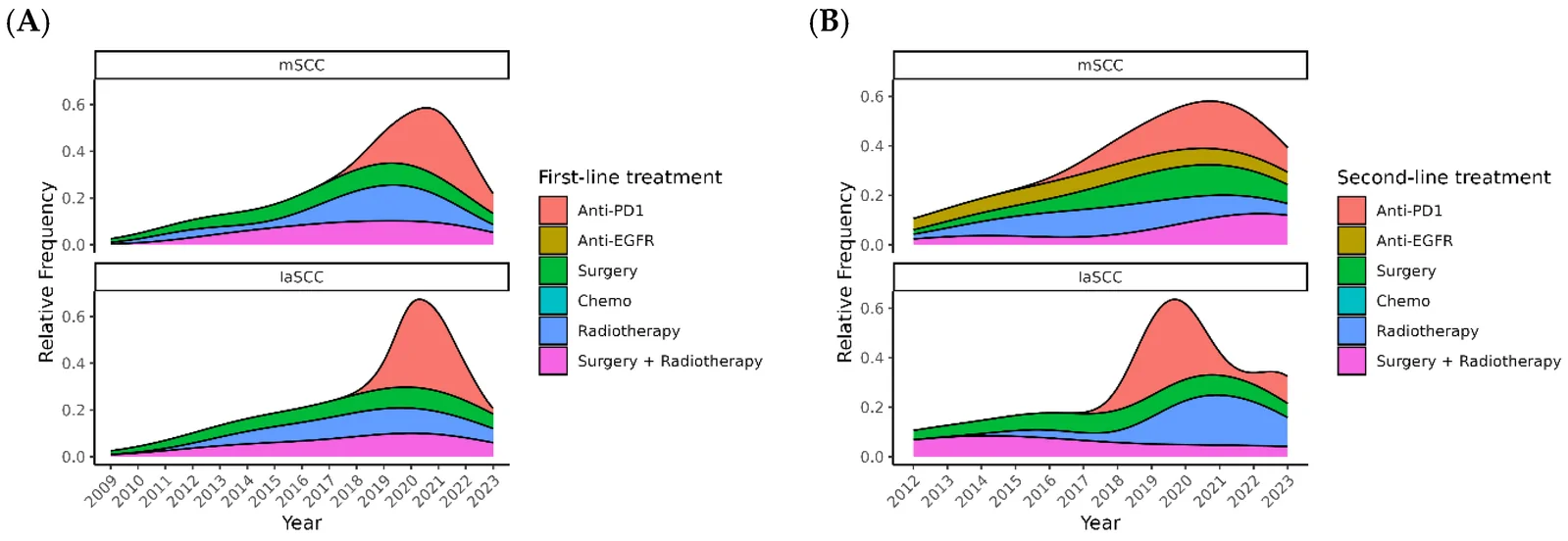
(**A**) Relative frequency of treatment modalities for the treatment of metastatic cSCC (mSCC) and locally advanced cSCC (laSCC) in the first-line and (**B**) second-line setting.

In the first-line setting, anti-PD1 was first used in 2017 or 2018 in the treatment of metastatic or locally advanced cSCC, respectively, with a rapid increase in its use thereafter (Figure 4A). Before 2017, the most common first-line treatment options were surgery and radiotherapy as single or combined modalities. In the second-line setting, anti-PD1 was first used one year earlier (Figure 4B). Before 2016, anti-EGFR was frequently used as second-line treatment in metastatic cSCC.

**Figure 5:**
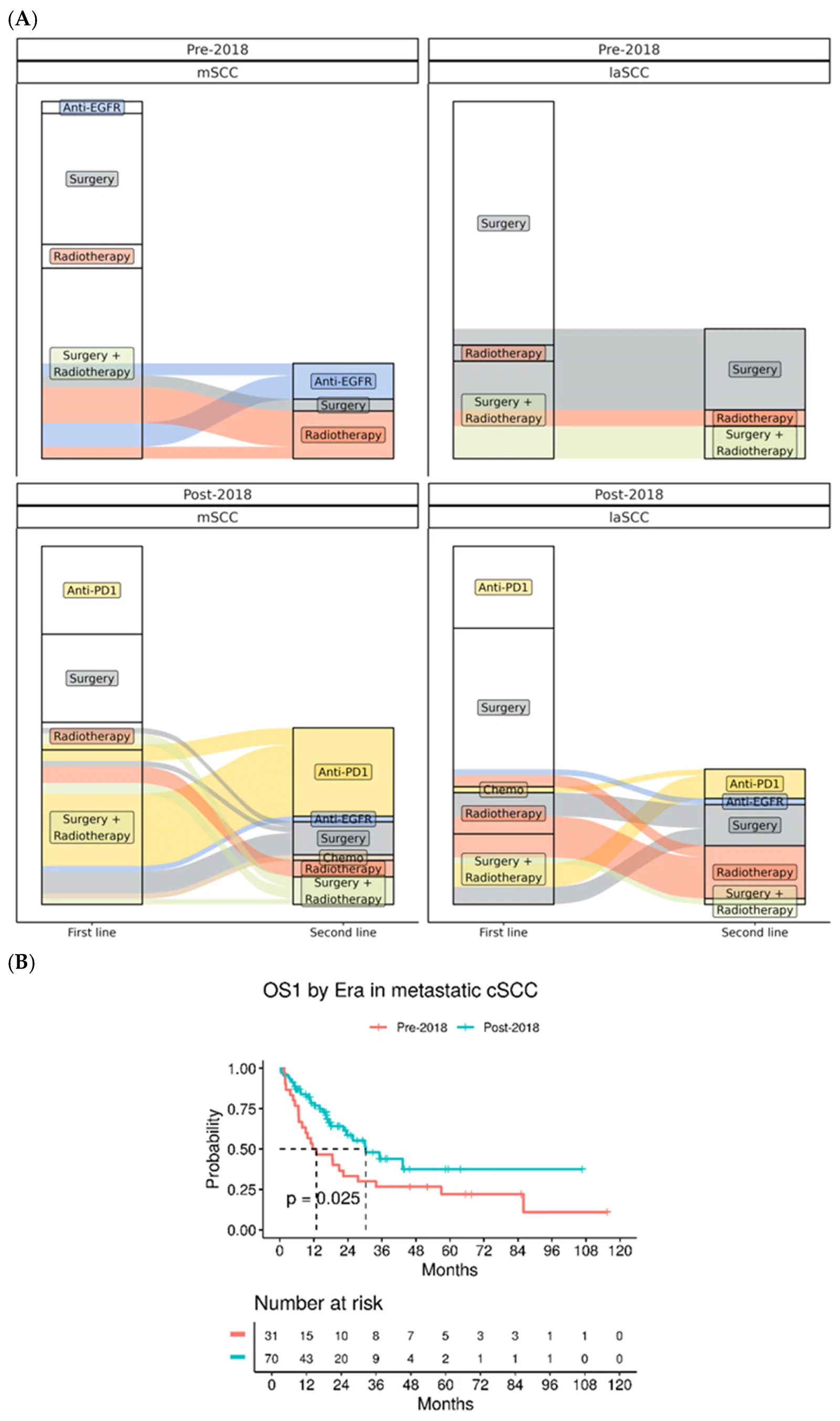
(**A**) Alluvial plot showing first-line and corresponding second-line treatments in patients with metastatic (mSCC) or locally advanced cSCC (laSCC) before and starting from 2018. (**B**) Overall survival (OS) for treatment in the pre- versus post-2018 era in mSCC. The 2018 cut-off marks the transition to regular use of anti-PD1 in the management of advanced cSCC at our centre.

Until 2018 (pre-2018 era), mSCC and laSCC were most commonly approached with surgery and radiotherapy (Figure 5A). For patients who progressed, second-line strategies in mSCC included anti-EGFR and radiotherapy, while in laSCC, surgery was frequently attempted (Figure 5A). After 2018, the therapeutic armamentarium included anti-PD1 both in first- and second-line (Figure 5A). Treatment in the post-2018 era was associated with a significantly longer OS for patients with mSCC (*p* = 0.025) (Figure 5B). The survival disadvantage of CLL patients compared to non-CLL patients (Figure 1) appeared more pronounced in the post-2018 era (Supplementary Figure S1), possibly indicating that CLL patients did not benefit from advances in systemic therapy to the same extent. Given the small number of CLL patients, this exploratory observation should be interpreted with caution and requires confirmation in larger cohorts.

**Figure 6:**
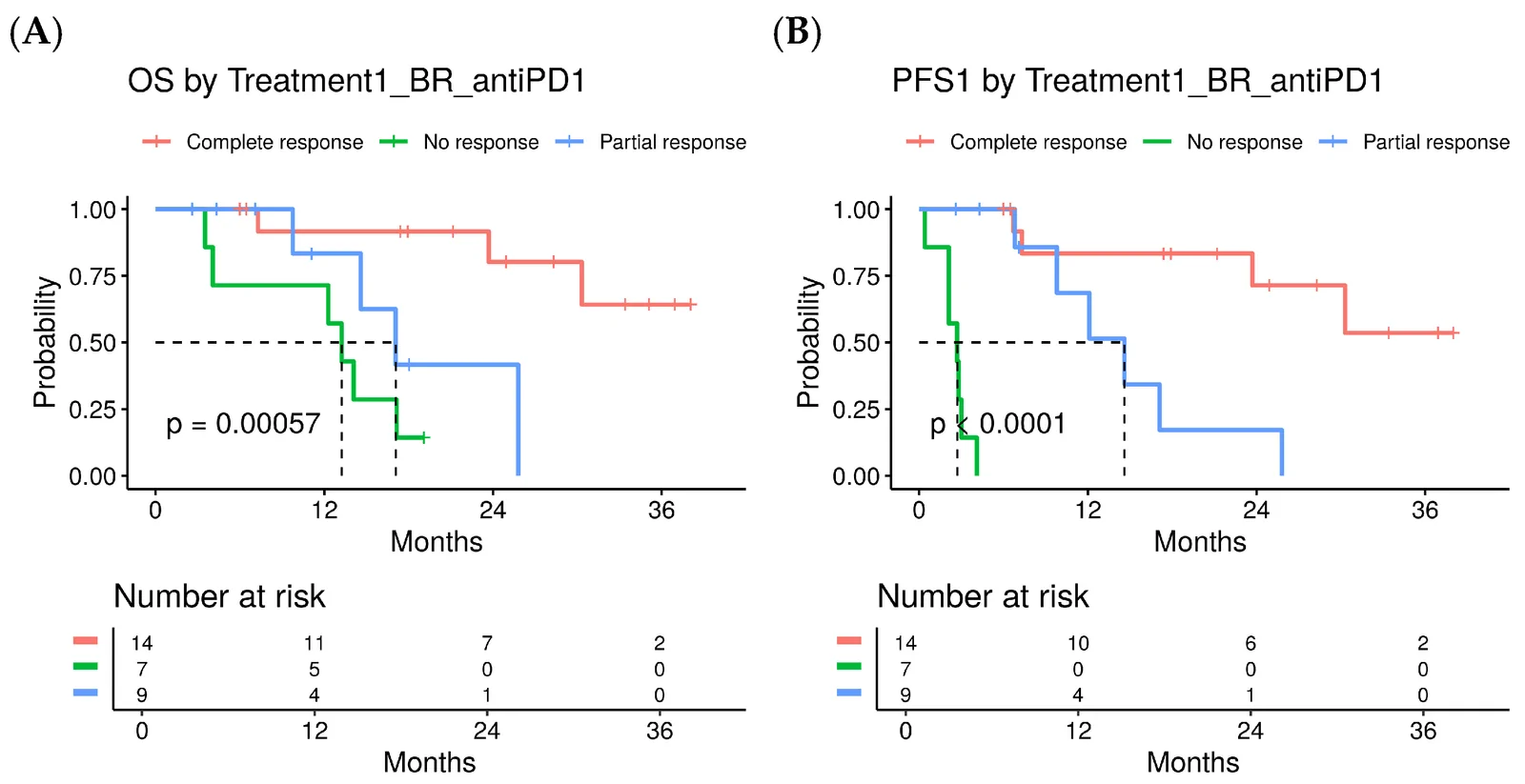
(**A**) Overall survival (OS) and (**B**) progression-free survival (PFS) for patients with advanced cSCC treated with first-line anti-PD1 according to best overall response (BOR).

Best overall response (BOR) to first-line anti-PD1 significantly correlates with OS: While patients with a complete response did not reach median OS (95% CI: 30.32-NR), patients with no response or a partial response had a median OS of 13.2 (95% CI: 4.07-NR) and 17.1 months (95% CI: 14.59-NR) (Figure 6A). Furthermore, patients with complete response as BOR had a 1-year PFS of 83.3% (95% CI: 64.7–100) and a 3-year PFS of 53.6% (95% CI: 26.9–100) (Figure 6B).

## 4. Discussion

This single-centre, retrospective study aimed to provide real-world insights into the management of advanced cSCC over the last decade, highlighting the evolving treatment landscape. As reported previously, advanced cSCC predominantly affects elderly men [16,17,19], and most patients had a history of extensive skin carcinogenesis, underscoring the need for balanced strategies that emphasize prevention and early detection while avoiding overtreatment. While the overall recurrence rate for primary cSCC is around 3–7% [20,21], in our cohort, over half of the cases originated from a primary cSCC—with a median time-lag of one year. Of these primary tumors, 65% progressed despite histologically confirmed complete resection, highlighting the need for better predictive markers of high-risk cases. Achieving clear margins nonetheless remains critical: patients whose primary cSCC was completely resected (R0) had significantly better OS than those with incomplete resection. A large retrospective study of unselected primary cSCC reported a complete excision rate of 90.9% [22]; in our cohort this rate was considerably lower, highlighting incomplete excision as a risk factor for disease progression.

The approval of the anti-PD1 antibody cemiplimab in mid-2019 marked a turning point in the treatment landscape of advanced cSCC. In our study, the first patients received first-line anti-PD1 in 2017 (mSCC) or 2018 (laSCC) through off-label and compassionate use programs. Previously, the mainstays of treatment were surgery and radiotherapy, frequently in combination, while anti-EGFR was commonly used in metastatic cSCC despite low and generally non-durable response rates (estimated at 26%) [23,24]. The broader adoption of ICI may have contributed to improved outcomes, as reflected by the prolonged OS after 2018 in our cohort of metastatic cSCC. (The cohort comprised all patients irrespective of treatment received.) Improvements in radiotherapy and surgical techniques likely also contributed. The need for increased multidisciplinary discussion and individualized treatment planning is reflected in the tripling of tumorboard presentations from 2018 onward.

Our data also highlight how locally advanced and metastatic cSCC are approached differently in the modern era. As metastatic cSCC most frequently shows loco-regional rather than distant spread, local treatment modalities such as complete surgical excision with or without adjuvant radiotherapy, or primary radiotherapy remain a cornerstone of first-line therapy alongside anti-PD1. In the second-line setting, anti-PD1 predominates in metastatic disease, whereas surgery or radiotherapy remain more common in locally advanced cSCC.

Underlying the shift to immunotherapy is the particular immunogenicity of cSCC. Given the high number of UV-induced mutations, cSCC is among the tumors with the highest mutational burden, which is associated with increased neoantigen formation and may contribute to its responsiveness to immune checkpoint inhibition [14,25]. Indeed, PD1 immune checkpoint blockade has proven very effective in the treatment of advanced cSCC in the registration trials [12,13], with comparable or higher overall response rates of 50–60% in real-world reports [16,17,18,26]. However, patients with immunosuppressive conditions such as chronic lymphocytic leukemia (CLL) or solid organ transplant recipients (SOTR) were largely excluded from the registration trials, and their outcomes remain less well characterized.

Patients with CLL develop cSCC more frequently, and these tumors show a more aggressive biological behavior compared to the general population [6,27]. This is thought to reflect cellular and humoral immune dysfunction, leading to impaired cancer cell recognition [28]. The largest CLL-specific response data come from an Australian real-world cohort of 286 patients with advanced cSCC, in which the response rate among 24 evaluable CLL patients was 50% compared to 64% in immunocompetent patients, with significantly poorer OS and PFS in the 88 immunocompromised patients overall (adjusted HR 1.8 for both) [26]. In our cohort, CLL patients received anti-PD1 at a rate comparable to the overall population, yet OS across all CLL patients was significantly reduced (12.9 vs. 35.1 months). While OS improved significantly for non-CLL patients with metastatic cSCC in the post-2018 era, the survival gap between CLL and non-CLL patients widened in our study (Supplementary Figure S1), further emphasizing the urgent need for prospective data and effective treatment strategies in this population.

In SOTR, varying degrees of iatrogenic immunosuppression over many years, depending on the organ transplanted, put these patients at very high risk of developing keratinocyte carcinomas, with aggressive cSCCs being much more frequent than in the general population [29,30]. In our study, SOTR with advanced cSCC showed a strongly reduced survival, with a median OS of less than 5 months. The use of ICI is often precluded in SOTR due to the high risk of transplant rejection [31], and only two of our 13 SOTR received anti-PD1. Kidney transplant recipients, the most common SOTR in our cohort, are particularly amenable to ICI due to the rescue possibility with hemodialysis, and the feasibility of anti-PD1 combined with pulsed corticosteroids has been shown [32]. The second most common SOTR in our cohort were heart and lung transplant recipients, where ICI requires extremely careful consideration, due to very high mortality in cases of ICI-induced rejection.

Anti-PD1 is increasingly being investigated in the perioperative setting. The recently published C-POST trial demonstrated that adjuvant cemiplimab significantly prolonged disease-free survival compared to placebo in patients with high-risk cSCC after macroscopic complete resection and postoperative radiotherapy (HR 0.32, *p* < 0.001) [33]. However, the efficacy of adjuvant anti-PD1 in incompletely resected cSCC remains to be established and warrants investigation in future trials. Complementing the adjuvant approach, neoadjuvant cemiplimab has shown promising results in resectable stage II–IV (M0) cSCC, with a pathological complete response rate of 51% after up to four doses prior to surgery [34]. Together, these perioperative strategies may help improve surgical outcomes and reduce recurrence, particularly in patients where complete resection is challenging.

These developments also raise the question of how immunosuppressed patients, who are routinely excluded from trials, should be incorporated into future studies. Since response-adapted de-escalation of surgery or radiotherapy [35] relies on a reliable and durable anti-PD1 response, and such data are lacking in immunosuppressed patients, these groups should be enrolled and analyzed as predefined strata rather than assumed to behave like immunocompetent patients. CLL patients can receive immune checkpoint inhibition without special adaptation and should be included in such trials, whereas SOTR require transplant-specific protocols and are better studied in dedicated settings.

The feasibility of such response-adapted strategies depends on how reliably treatment response predicts long-term benefit. In our cohort, patients achieving a complete response under anti-PD1 had a 1-year progression-free survival of 83.3%, comparable to the reported frequency of ongoing responses of 87.8% at 1 year [13]. However, relapses do occur in this subgroup: at 3 years, only 53.6% survived without disease progression. Good biomarkers for patient stratification are lacking, but may in the future aid in decision making regarding treatment continuation after complete response.

Limitations of this study include the single-centre, retrospective design and the low number of patients in some subgroups such as SOTR and CLL, restricting generalizability of the findings. Future multicentre studies or meta-analyses would help extend our findings. As advanced cSCC may occasionally be discussed primarily at the separate head and neck tumorboard, we cannot exclude that a small number of cases were not captured by our search. Overall survival may not fully reflect cancer-specific outcomes in this elderly, comorbid population; disease-specific survival would be more informative, but reliable cause-of-death data were not available in our retrospective setting. Finally, besides the introduction of anti-PD1 immunotherapies, medical treatment has evolved rapidly over the study period, which may introduce additional variability in treatment outcomes.

This study contributes to the understanding of real-world treatment of advanced cSCC. It emphasizes the dynamics over the last decade, in particular the introduction of immune checkpoint blockade, but also highlights the unmet clinical need in patients with CLL and in SOTR. Future research should focus on high-risk patient populations and on optimal treatment sequences in locally advanced and metastatic cSCC.

## Figures and Tables

**Table 1:** Overview over complete cohort.

	Total
	(N = 189)
**Age (at diagnosis of advanced)**	
Mean (SD)	78.4 (11.8)
Median [Min, Max]	79.0 [37.0, 99.0]
**Sex**	
Male	137 (72.5%)
Female	52 (27.5%)
**Organ transplantation**	
None	176 (93.1%)
Kidney	5 (2.6%)
Heart	4 (2.1%)
Lung	4 (2.1%)
**Comorbidities**	
None	152 (80.4%)
NHL	13 (6.9%)
CLL	12 (6.3%)
Hematological malignancy	8 (4.2%)
Chronic viral infection	3 (1.6%)
Xeroderma pigmentosum	1 (0.5%)
**Previous AK**	
5+	86 (45.5%)
None	74 (39.2%)
1–4	29 (15.3%)
**Previous cSCC**	
None	80 (42.3%)
1–4	69 (36.5%)
5+	40 (21.2%)
**Previous BCC**	
None	100 (52.9%)
1–4	57 (30.2%)
5+	32 (16.9%)

**Table 2:** Patients with primary non-advanced cSCC.

	Total
	(N = 100)
**Age (at diagnosis of primary tumor)**	
Mean (SD)	76.4 (10.1)
Median [Min, Max]	77.0 [43.0, 94.0]
**Localization**	
Face	63 (63.0%)
Scalp	18 (18.0%)
Ear	8 (8.0%)
Extremities	4 (4.0%)
Trunk	2 (2.0%)
Neck	2 (2.0%)
Missing	3 (3.0%)
**Risk group localization**	
Low risk	62 (62.0%)
High risk	35 (35.0%)
Missing	3 (3.0%)
**Thickness (mm)**	
Mean (SD)	5.43 (3.96)
Median [Min, Max]	4.00 [1.00, 18.0]
**Perineural invasion**	
Not reported	76 (76.0%)
Yes	9 (9.0%)
No	15 (15.0%)
**Treatment**	
Surgery	85 (85.0%)
Surgery followed by radiotherapy	8 (8.0%)
Cryotherapy or other destructive method	5 (5.0%)
Radiotherapy	2 (2.0%)
**Time to advanced cSCC (months)**	
Mean (SD)	20.5 (25.4)
Median [Min, Max]	12.2 [0.8, 132]

**Table 3:** Patients with locally advanced cSCC.

	Total
	(N = 86)
**Diameter of laSCC (mm)**	
Mean (SD)	49.7 (35.5)
Median [Min, Max]	36.0 [12.0, 150]
Missing	46 (53.5%)
**Thickness of laSCC (mm)**	
Mean (SD)	15.1 (14.2)
Median [Min, Max]	12.5 [1.00, 80.0]
Missing	48 (55.8%)
**Treatment**	
Surgery	42 (48.8%)
Surgery + Radiotherapy	19 (22.1%)
Anti-PD1	14 (16.3%)
Radiotherapy	9 (10.5%)
Other	1 (1.2%)
Chemo	1 (1.2%)

**Table 4:** Patients with metastatic cSCC.

	Total
	(N = 103)
**Localization of metastases**	
Regional (in-transit and/or lymph node metastases)	95 (92.2%)
Distant	8 (7.8%)
**First-line treatment**	
Surgery	27 (26.2%)
Surgery + Radiotherapy	45 (43.7%)
Anti-PD1	19 (18.4%)
Radiotherapy	7 (6.8%)
Other	4 (3.9%)
Anti-EGFR based	1 (1.0%)
**Second-line treatment**	
Anti-PD1	16 (15.5%)
Surgery	7 (6.8%)
Radiotherapy	7 (6.8%)
Surgery + Radiotherapy	5 (4.9%)
Anti-EGFR based	4 (3.9%)
Other	3 (2.9%)
Anti-PD1 + Anti-EGFR	1 (1.0%)
Chemo	1 (1.0%)
Missing	59 (57.3%)

## Data Availability

The data presented in this study are available on request from the corresponding author. The data are not publicly available due to privacy restrictions and the conditions of the ethics approval.

## Supplementary Materials

The following supporting information can be downloaded at: https://www.mdpi.com/article/10.3390/curroncol33080449/s1. Figure S1: Overall survival (OS) for patients with CLL and mSCC in the post-2018 era; Table S1: Comparison of baseline characteristics between patients presenting in the pre-2018 and post-2018 era. *p*-values from Wilcoxon rank-sum test for continuous and Fisher’s exact test for categorical variables.
