# Supplementary material for: Management of Advanced Cutaneous Squamous Cell Carcinoma over the Last Decade: A Single-Centre Retrospective Study
Source: Curr Oncol. 2026 Jul 27;33(8):449. doi: 10.3390/curroncol33080449 (PMC13510792; doi:10.3390/curroncol33080449)
Supplement: Supplementary file 1 [file curroncol-33-00449-s001.zip › curroncol-4372337-supplementary.pdf]

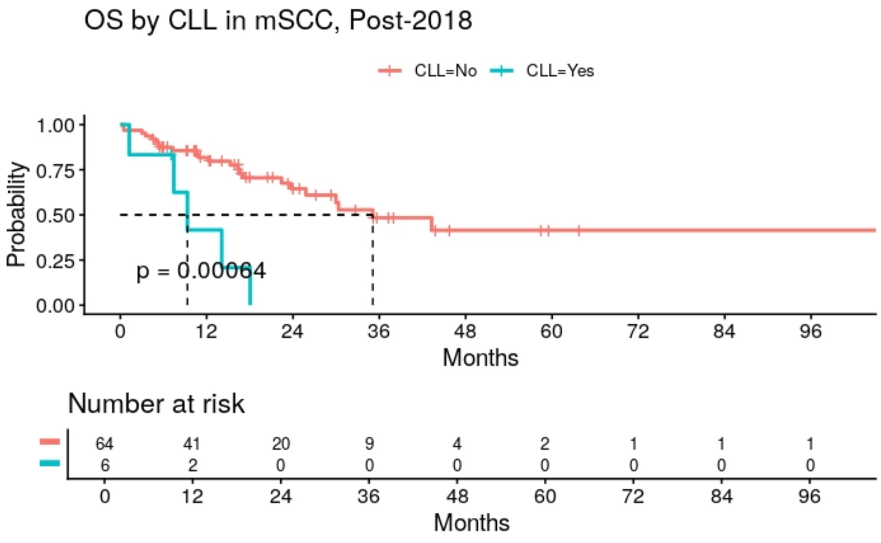

Figure S1. Overall survival (OS) for patients with CLL and mSCC in the post-2018 era.

Table S1. Comparison of baseline characteristics between patients presenting in the pre-2018 and post-2018 era. P-values from Wilcoxon rank-sum test for continuous and Fisher's exact test for categorical variables.

	Pre-2018 (N=56)	Post-2018 (N=133)	p-value
**Age (at diagnosis of advanced)**			
Mean (SD)	76.3 (13.5)	79.2 (11.0)	0.148
Median [Min, Max]	77.0 [45.0, 99.0]	81.0 [37.0, 96.0]	
**Sex**			
Male	38 (67.9%)	99 (74.4%)	0.376
Female	18 (32.1%)	34 (25.6%)	
**Advanced cSCC category**			
mSCC	32 (57.1%)	71 (53.4%)	0.749
laSCC	24 (42.9%)	62 (46.6%)	
**Organ transplantation**			
No	50 (89.3%)	126 (94.7%)	0.21
Yes	6 (10.7%)	7 (5.3%)	
**CLL**			
No	50 (89.3%)	127 (95.5%)	0.187
Yes	6 (10.7%)	6 (4.5%)	
**Previous cSCC**			
None	31 (55.4%)	49 (36.8%)	0.0705
1 - 4	16 (28.6%)	53 (39.8%)	
5+	9 (16.1%)	31 (23.3%)	
**Previous AK**			
5+	26 (46.4%)	60 (45.1%)	1
None	22 (39.3%)	52 (39.1%)	
1 - 4	8 (14.3%)	21 (15.8%)	
**Documented preceding primary cSCC**			
Yes	32 (57.1%)	68 (51.1%)	0.524
No	24 (42.9%)	65 (48.9%)	
